# Imprisoning Yoga: Yoga Practice May Increase the Character Maturity of Male Prison Inmates

**DOI:** 10.3389/fpsyt.2019.00406

**Published:** 2019-06-13

**Authors:** Nóra Kerekes, Sven Brändström, Thomas Nilsson

**Affiliations:** ^1^Department of Health Sciences, University West, Trollhättan, Sweden; ^2^Clinical Associate of the Center for Well-Being, Washington University School of Medicine, St. Louis, MO, United States; ^3^Centre for Ethics, Law and Mental Health (CELAM), Institute of Neuroscience and Physiology, University of Gothenburg, Gothenburg, Sweden

**Keywords:** character maturity, prison, self-directedness, temperament and character inventory, yoga

## Abstract

**Background:** A specific personality profile, characterized by low character maturity (low scores on the self-directedness and cooperativeness character dimensions) and high scores on the novelty seeking temperament dimension of the temperament and character inventory (TCI), has been associated with aggressive antisocial behavior in male prison inmates. It has also been shown that yoga practiced in Swedish correctional facilities has positive effects on the inmates’ well-being and on risk factors associated with criminal recidivism (e.g., antisocial behavior). In this study, we aimed to investigate whether the positive effect of yoga practice on inmates’ behaviors could be extended to include eventual changes in their personality profile.

**Methods:** Male prison inmates (*N* = 111) in Sweden participated in a randomized controlled 10-week long yoga intervention trial. Participants were randomly assigned to either a yoga group (one class a week; *n* = 57) or a control group (free of choice weekly physical activity; *n* = 54). All the inmates completed the TCI questionnaire before and after the intervention period as part of an assessment battery.

**Results:** After the 10-week-long intervention period male inmates scored significantly lower on the novelty seeking and the harm avoidance and significantly higher on the self-directedness dimensions of the TCI. There was a significant medium strong interaction effect between time and group belonging for the self-directedness dimension of character favoring the yoga group.

**Conclusion:** A 10-week-long yoga practice intervention among male inmates in Swedish correctional facilities increased the inmates’ character maturity, improving such abilities as their capability to take responsibility, feel more purposeful, and being more self-acceptant—features that previously were found to be associated with decreased aggressive antisocial behavior.

## Introduction

### Personality and Trait Aggression of Prison Inmates

A long-standing question among scientists is whether there is a specific personality profile associated with criminality. Hitherto, no personality profile characterizing all criminals has been identified, although some specific personality constellations may apply to many criminals (1). Generally, extreme temperament constellations in combination with low or very low character maturity have been associated with criminal and aggressive antisocial behaviors (2–4).

According to Cloninger’s psychobiological model of personality, the temperament dimensions (comprising the dimensions novelty seeking, harm avoidance, reward dependence, and persistence) of an individual’s personality are neurobiologically based, inheritable features (5, 6). These temperament dimensions are defined as habitual responses to stimuli and are considered to be rather stable over the individual’s lifetime, although they might interact with and be modified by the character dimensions (comprising the dimensions self-directedness, cooperativeness, and self-transcendence) of an individual’s personality (7, 8).

Among the temperament dimensions, high scores on novelty seeking, in particular, have been found to be associated with aggressive and antisocial behaviors in prison inmates (4). This temperament dimension captures exploratory activity in response to novel stimuli, impulsive decision making, and temper outbursts, responses which are highly consistent with aggressive and antisocial acts (5). The second most often recognized increased temperament dimension in prison inmates was found on the harm avoidance dimension. This temperament profile expresses pessimistic, fearful, and doubtful worrying actions, which are highly correlated with self-directed aggression, increased prevalence of self-harm and suicidal behaviors in inmates (4).

The character dimensions, self-directedness, cooperativeness, and self-transcendence, are based on social learning, and are therefore expected to mature over time (5, 8). Low scores on self-directedness and cooperativeness, indicating immaturity, low self-governance, social incompetence or intolerance, uncooperativeness, and revengefulness, have repeatedly been associated with mental ill-health (5, 9–12), also in forensic populations (13), and with aggressive and antisocial behaviors in inmates (4).

Actually, extremes in the temperamental dimensions in combination with low character maturity predict psychiatric ill health and aggressive antisocial behavior. However, a high level of character maturity has been found to be a protective factor (14). Consequently, an extreme temperament is not an unambiguous sign of mental illness or deviant behavior when found in combination with a normal to high level of character maturity. These findings suggest that interventions that increase an individual’s character maturity can mitigate the effect of extreme temperaments, decrease aggression, antisocial behavior, and different aspects of psychiatric ill health.

### Positive Effects of Yoga on Prison Inmates’ Behavior and Mental Health

In recent years, yoga has become popular in many correctional institutions around the world as a complementary rehabilitation tool offered to inmates. An increasing number of studies show that regular yoga practice is associated with an increased level of impulse control (15), attenuation of anger, aggression (16), and antisocial behaviors (15), and with a significantly decreased level of paranoid ideations (17), each of these phenomena being a key variable related to criminal behavior. Furthermore, improvements on variables that can increase offenders’ abilities to participate in treatments have also been observed, for example, that yoga significantly can increase positive and decrease negative emotional states (15, 18); sustain attention (15); decrease depression, anxiety, and obsession (17, 19); and reduce the individual’s experienced stress level (15).

Neurobiological changes, including modulation of neurotransmitters (increased serotonin, decreased cathecholamines), may explain the above-mentioned phenotypical changes (20, 21), suggesting improvement of cortical controls, and improved social functioning.

Based on this notion of behaviorally induced modification of the neurobiological conditions that regulate decision making, we hypothesize that regular yoga practice can induce neurobiological changes in participants, and that these changes can be phenotypically measured in the form of an improvement in the level of character maturity.

The aim of the present study is therefore to investigate within correctional settings the effect of 10 weeks of yoga practice on male prison inmates’ temperament and character profiles.

## Methods

### Procedures

For a detailed description of the study procedure and extended information on the participants, please see Ref. (15).

Briefly summarized: data collection was done from November 2013 to July 2015 in seven Swedish high- and medium-security male correctional facilities.

The participants completed a pre-intervention assessment (time 1), which included the Temperament and Character Inventory-Revised 140 (TCI-R140) (see below for a detailed description) and other self-report questionnaires, before being randomly assigned either to yoga classes or to a waiting list (the control group). During the 10-week intervention period, the participants in the yoga group attended a 90 minutes yoga class once a week. The yoga class was led by prison officers trained by the Swedish Prison and Probation Service in a yoga program specifically designed for inmates (Krimyoga). The participants in the control group were asked to perform some other type of physical activity for 90 minutes each week during the 10-week period, during which they were on a waiting list to participate in yoga classes. Upon completion of the 10-week period, the participants in both groups, i.e., the yoga group and the control group, once again completed the TCI-R140 as part of the post-intervention assessment (time 2), where they also reported the amount of weekly physical activity.

### Participants

The study sample included 201 male volunteers. Their age ranged from 18 to 62 years. Sixty-eight (33.8%) individuals of the original sample left the study for different reasons, such as own request, being transferred to other correctional facilities, misconduct, and so on (**Table 1**). Of the remaining inmates (133), 67 completed the study within the yoga group and 66 in the control group. Due to the absence of numerous items in their TCIs (more than 5% missing), 22 participants were excluded from the present analyses, leaving 57 participants in the yoga group and 54 in the control group for which TCI data were evaluated.

**Table 1:** Attrition rates and reasons for attrition in the yoga and control groups.

	Number (%)		p
	Yoga group (n = 121)	Control group (n = 80)	
**Attrition rate**	54 (44.6%)	14 (17.5%)	<0.001
**Reason for attrition**			
Participant’s request	10 (18.5%)	6 (42.9%)	0.08
Transfer	12 (22.2%)	2 (14.3%)	0.72
Misconduct	5 (9.3%)	0 (0%)	0.58
Illness, injury, or mental health problems	7 (13%)	1(7.1%)	1.00
Did not attend all yoga classes	4 (7.4%)	–	–
Discontinuous yoga classes (personal)	2 (3.7%)	–	–
Yoga class interfered with school or work	4 (7.4%)	–	–
Chose yoga outside the study	–	1 (7.1%)	–
Not collected data	6 (11.1)	3 (21.4%)	0.38
Not specified	4 (7.4%)	1 (7.1%)	1.00

As depression level could strongly affect different dimensions of the personality profile (such as harm avoidance and self-directedness), it is important to establish whether the study design (randomization) resulted in a somewhat equal variance with regard to depressive symptoms in the two groups. However, it was not possible to merge data files with any clinical data (e.g., inpatient data). Instead, we used the self-reported level of psychological distress (see publication 17) to compare the two groups on the depression primary symptom dimension. There was no significant difference (*p* = 0.76) in self-rated depressive symptoms between the yoga group (M = 0.99; SD = 0.08) and the control group (M = 1.04; SD = 0.10).

### Measure

#### TCI-R 140

The TCI is a self-report personality questionnaire based on Cloninger’s psychobiological model of temperament and character (5). There are several different versions of the TCI (e.g., TCI-240, TCI-125, and TCI-R), of which we used one of the latest versions, the TCI-R140, which is a short and revised version of the original TCI-240 inventory. The TCI-R140 contains 136 items covering the four temperament dimensions (i.e., novelty seeking, harm avoidance, reward dependence, and persistence) and the three character dimensions (i.e., self-directedness, cooperativeness, and self-transcendence). The remaining four items are built in as control questions. Each dimension contains 20 items, with the exception of self-transcendence, which has only 16 items (22). Each question is rated on a five-point Likert scale ranging from 1 (definitely false) to 5 (definitely true) (23). The Swedish version of the TCI has been validated (8), showing good internal consistency and factor structure and high test–retest reliability. The TCI-R140, the short version of the TCI-R, has also been found to have good reliability coefficients and factor structures even across cultures (24, 25). Internal reliability of each dimension in the present study was acceptable and varied between Cronbach alphas of .76 (novelty seeking) and .87 (self-directedness).

### Statistical Analyses

The data did not violate the assumption of normality. The significance level was set at *p* < 0.05. The scores are presented by mean (M) and standard deviation (SD). Internal reliability of each dimension of TCI was presented with Cronbach alpha. Comparison of attrition rate between the groups was calculated with Fisher’s exact test. A two-way repeated-measures ANOVA was used with two factors (time 1 and time 2) and with “group” as between subject’s variable. Effect size is calculated as the Eta^2^, where .01 signals a small, .06 a medium, and .14 a large effect (26).

### Ethical Considerations

The study was approved by the regional Ethical Review Board in Linköping (2013/302-31). The prison inmates interested in voluntary participation received both verbal and written information about the study procedure and conditions of participation. Written informed consent was obtained from all participants. Upon study completion, the participants received a phone card valued at 200 Swedish crowns (about 20 euros).

## Results

**Figure 1:**
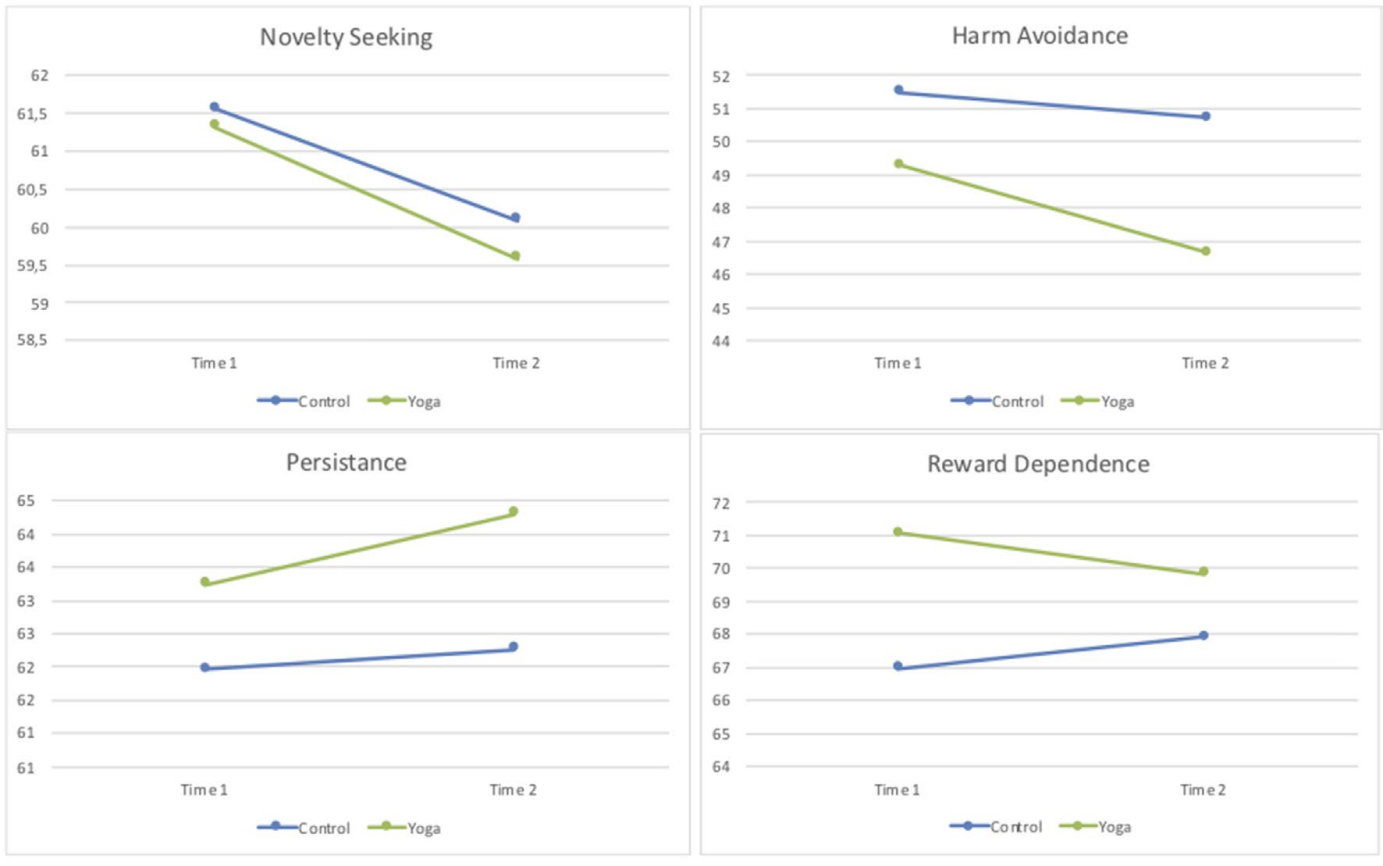
Mean scores of the measures in each temperament dimension of temperament and character inventory (TCI) in control and yoga groups at pre- and post-intervention (time 1 and time 2).

**Figure 2:**
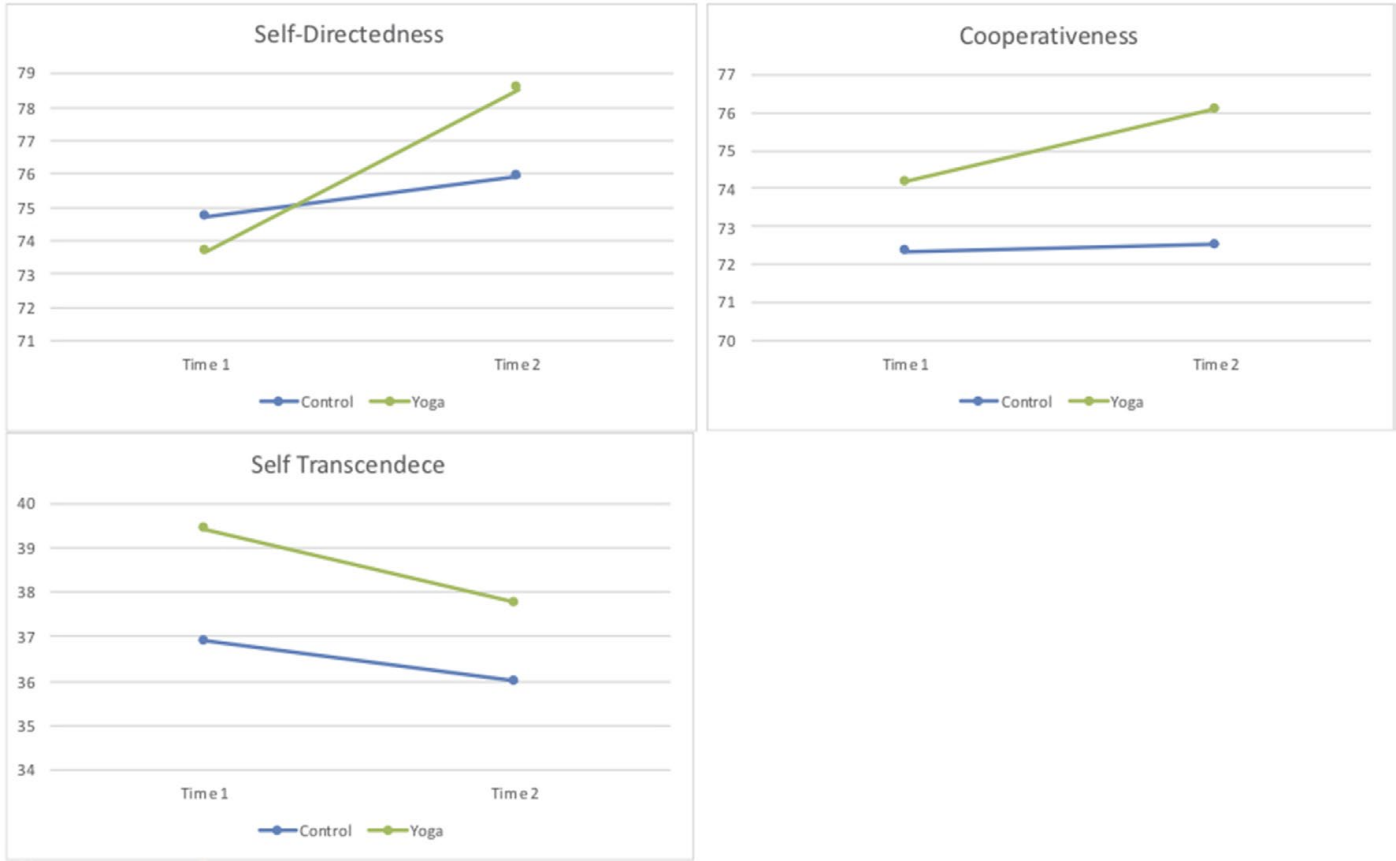
Mean scores of the measures in each character dimension of TCI in the control and yoga groups at pre- and post-intervention (time 1 and time 2).

The mean scores of the seven dimensions of the TCI at pre- and post-intervention for the yoga group and for the control group are presented in **Table 2** and **Figures 1** and **2**.

**Table 2:** Average ratings at pre- and post-intervention assessment (time 1 and time 2) in the yoga and control groups.

TCI dimensions	Yoga group (n = 57), M (SD)		Control group (n = 54), M (SD)	
	Time 1	Time 2	Time 1	Time 2
**Temperament dimensions**				
Novelty seeking	61.3 (11.4)	59.6 (10.2)	61.6 (10.6)	60.1 (10.1)
Harm avoidance	49.3 (13.9)	46.7 (11.6)	51.5 (11.8)	50.7 (12.8)
Reward dependence	63.2 (12.8)	64.3 (11.8)	62.0 (11.0)	62.3 (10.4)
Persistence	71.1 (12.2)	69.8 (11.5)	67.0 (12.0)	67.9 (12.1)
**Character dimensions**				
Self-directedness	73.7 (14.4)	78.5 (14.0)	74.7 (13.3)	75.9 (14.3)
Cooperativeness	74.2 (11.3)	76.1 (10.6)	72.4 (9.9)	72.5 (10.6)
Self-transcendence	39.4 (11.6)	37.8 (12.2)	36.9 (11.6)	36.0 (12.1)

TCI, temperament and character inventory.

Repeated-measures ANOVA was conducted to assess the impact of the yoga intervention compared to the control group of physical activity on participants’ personality traits according to TCI across two periods (pre-intervention and post-intervention). There was a significant interaction between treatment type and time, for the character dimension self-directedness [*F*(1,109) = 4.88, *p* = 0.029, partial Eta^2^ = 0.04], in favor of the yoga group. There were also univariate within-subjects effects for time for the personality dimensions of novelty seeking [*F*(1,109) = 6.09, *p* = 0.015, partial Eta^2^ = 0.05], harm avoidance [*F*(1,109) = 4.46, *p* = 0.037, partial Eta^2^ = 0.04], and for the character dimension of self-directedness [*F*(1,109) = 13.43, *p* < 0.001, partial Eta^2^ = 0.11]. No significant effects were seen for the remaining personality dimensions of TCI (data upon request).

## Discussion

To the best of our knowledge, the present study is the first to investigate the effect of 10 weeks of regular yoga practice on personality traits, operationalized in terms of temperament, and character profiles, of male prison inmates. According to our results, the male inmates showed significant changes during the 10 weeks of study period in the novelty seeking, harm avoidance, and self-directedness dimensions of their personality profile. Also, we have detected a significant group × time interaction in the dimension of self-directedness in support of the yoga intervention. The importance of these findings lies in their support of the previously suggested hypothesis that increased character maturity (self-directedness and cooperativeness) may attenuate inmates’ deviant behaviors and mental health problems, thereby having a positive effect on the criminogenic factors of the affected individuals and possibly preventing them from criminal reoffending.

To measure changes in the personality profiles that are of relevance for antisocial behaviors, it is necessary to use a personality measure that is based on developmental theories covering neurobiological, psychosocial, cognitive, and personality aspects. Cloninger’s temperament and character model with its accompanying questionnaire provides a valuable tool for this purpose (27). This psychobiological model of personality states that the character component of an individual’s personality changes over time, due to social learning and cognitive maturation. This process is mirrored in the ability to identify the self as an autonomous, purposeful, and in relation to the environment, dynamically integrated individual. These attributes are measured by the self-directedness and cooperativeness dimensions in the TCI (5), which are supposed to encompass the ability to control extreme temperamental variations and to deal with and overcome both external and internal constraints, such as reaction patterns, abilities, and drives. According to the original theory, the character dimensions change and maturate due to learning and self-insight processes during adolescence (28), whereas the temperament dimensions are stable and inherited. However, the results of a Japanese research group support the opposite hypothesis, namely that genetic factors play a more important role in the development of character (29, 30). The most recent twin studies offer a “compromise,” stating that genetic and shared environmental factors account for a substantial amount of the inter-individual variation in both temperament and character (10). Based on this kind of finding, the present study investigated whether 10 weeks of yoga practice, compared to regular physical activity, can affect the temperament and character dimensions of personality.

Our results clearly indicate that 10 weeks of physical activity (including yoga) had a positive effect on the temperament dimension of novelty seeking in male inmates. That physical activity, and yoga specifically, attenuates inmates’ impulsive behavior has previously been shown using a computer-based measure of impulse control (15). These findings, i.e., decreases in impulsive decision making and in loss of temper (high novelty seeking), suggest that there is an improvement of the cortical controls in the brain that are responsible for inhibition of the limbic drives.

In the yoga group a prominent decrease of harm avoidance was detected while the scores in this temperament dimension also decreased in those performing free choice of physical activity. It is in this context important to state that there was no significant difference in the level of depression between participants in the yoga and control groups at preintervention, since depression is a state that strongly could affect ratings on this dimension. Harm avoidance captures worry, fear of uncertainty, and fatigability (27), which can be mirrored in negative deactivated affects (e.g., being scared, bored, tired, ashamed) or in the anxiety, phobic anxiety, and paranoid ideation, which all are primary symptom dimensions of psychological distress. In agreement with our result, showing that yoga specifically decreases the scores on the personality dimension of harm avoidance, it has been shown that yoga-practicing prison inmates also report significantly less negative deactivated affects (15), and that yoga significantly decreases anxiety and phobic anxiety, specifically the distress level expressed as paranoid ideation (17).

Our results also show that the scores on the cooperativeness character dimension increased among the inmates in the yoga group, however this change did not reach significance. A plausible interpretation of the increase in cooperativeness, at least for prison inmates within the Swedish Prison and Probation Service, can be that organized activities, such as those studied improve inmates’ social consciences and possible also their social acceptance. In other words, yoga practice may, in medium- and high-security prison settings, be a group activity that could improve inmates’ compassion, compliance, and social flexibility/tolerance.

The most prominent and interesting result is that there was an interaction effect of time and group, proving that the self-directedness character dimension improved in the inmates who had participated in yoga classes; a significant improvement with an effect that was moderate. In our study, as in other studies on personality profiles in prison samples (4, 31, 32), inmates were generally found to have low character maturity. Any improvement in their character maturity is likely to enhance their ability to control impulses and reactions originating from their extreme temperaments. That is why a significant improvement in the character dimension of self-directedness is so important.

Yoga is a physical exercise that also includes training of the mind. During the different yoga poses participants are encouraged to focus on themselves and their breathing, and to observe their own body (e.g., posture, muscle contraction or relaxation) and the information it provides (e.g., tension, pain, warmth or cold). Yoga practice has been coupled to neurochemical changes, such as decreased production of cortisol and adrenalin and boosted production of serotonin and melatonin (21), which can be associated with improved well-being and enriched self-esteem. These neurochemical changes most probably explain the changes in the personality dimensions found in this study, i.e., the enhanced levels of self-acceptance and resourcefulness found in the inmates who had participated in regular yoga training. This represents a significant improvement of the level of self-determination, coupled with an increased level of responsibility and purposefulness, which are important qualities when it comes to an individual’s ability to adapt in a prosocial way. This is exactly the kind of ability that inmates are in need of upon release from correctional facilities and when meeting and managing the challenges related to the process of reintegration into society.

## Strengths and Limitations

This study has both strengths and limitations. To our knowledge, the study is the biggest randomized controlled trial (RCT) within a correctional setting to have investigated the effects of a 10-week yoga intervention on personality aspects. The randomized controlled design, and its considerable number of participants, gives strength to the study. A further strength is the use of a well-validated psychobiological model of personality and its accompanying questionnaire, which has been shown to measure changes in personality dimensions over time.

One obvious limitation of the study is that we only focused on the male inmates’ personality profiles. As personality profiles are strongly gender-specific, we did not attempt to merge data for male and female inmates. Moreover, we were unable to perform separate analyses for female inmates due to the low number of female participants in the original study (15). Consequently, we lack knowledge about the effects of yoga on females in prison settings. Another limitation of the study is the absence of information about the clinical and demographic characteristics of the participants. However, such eventual bias would most probably have been ruled out by the rigorous study design (RCT), as demonstrated with the example of self-rated depressive symptoms. In addition, the attrition rate was larger in the yoga group fostering some suspicions that individuals not benefiting from this intervention ended their participation. However, when comparing available reasons for termination no significant difference was found between the two groups.

The sole reliance on self-reported data is also a limitation, as is the pre- and post-design without a longer follow-up period reflecting the stability of the gained effects.

## Conclusion

The present study is the first to provide evidence of the positive effects of regular yoga exercise for male prison inmates in terms of the personality dimension scores measuring the inmates’ character maturity. These results show that yoga can strengthen the inmates’ self-acceptance, purposefulness, and sense of responsibility, which are qualities that promote a more peaceful and safer environment in the correctional settings, and that also provide a foundation for the development of a prosocial lifestyle upon release. However, despite the promising nature of these results, they must be investigated and confirmed in future studies before we can draw any definitive conclusions.

## Data Availability Statement

The datasets generated for this study are available on request to the corresponding author.

## Ethics Statement

The study was approved by the regional Ethical Review Board in Linköping (2013/302-31). The prison inmates interested in voluntary participation received both verbal and written information about the study procedure and conditions of participation. Written informed consent was obtained from all participants. Upon study completion, the participants received a phone card valued at 200 Swedish crowns (about 20 euros).

## Author Contributions

NK designed and led the yoga study. She performed the data analyses and was responsible for writing the manuscript. SB contributed with coding the TCI raw data, statistic help, and in writing the manuscript. TN contributed to the manuscript with statistical help, critically important intellectual feedback on interpretation of our results as well as on writing the manuscript.

## Funding

The Swedish Prison and Probation Service has financed the present study, which had a project title “Psykobiologiska effekter av yoga i anstaltmiljö” and project number 2012-251.

## Conflict of Interest Statement

NK was employed at the Swedish Prison and Probations Services, R&D, during data assessment. The remaining authors declare that the research was conducted in the absence of any commercial or financial relationships that could be construed as a potential conflict of interest.
